# Humidity-dependent electrical performance of CuO nanowire networks studied by electrochemical impedance spectroscopy

**DOI:** 10.3762/bjnano.14.54

**Published:** 2023-06-05

**Authors:** Jelena Kosmaca, Juris Katkevics, Jana Andzane, Raitis Sondors, Liga Jasulaneca, Raimonds Meija, Kiryl Niherysh, Yelyzaveta Rublova, Donats Erts

**Affiliations:** 1 Institute of Chemical Physics, University of Latvia, 19 Raina Blvd., LV-1586, Riga, Latvia; 2 Faculty of Chemistry, University of Latvia, 19 Raina Blvd., LV-1586, Riga, Latvia

**Keywords:** CuO, electrochemical impedance spectroscopy, humidity, nanowire, sensor

## Abstract

Electrochemical impedance spectroscopy was applied for studying copper oxide (CuO) nanowire networks assembled between metallic microelectrodes by dielectrophoresis. The influence of relative humidity (RH) on electrical characteristics of the CuO nanowire-based system was assessed by measurements of the impedance *Z*. A slight increase of *Z* with increasing RH at low humidity was followed by a three orders of magnitude decrease of *Z* at RH above 50–60%. The two opposite trends observed across the range of the examined RH of 5–97% can be caused by water chemisorption and physisorption at the nanowire interface, which suppress electronic transport inside the p-type semiconductor nanowire but enhance ionic transport in the water layers adsorbed on the nanowire surface. Possible physicochemical processes at the nanowire surface are discussed in line with equivalent circuit parameters obtained by fitting impedance spectra. The new investigation data can be useful to predict the behavior of nanostructured CuO in humid environments, which is favorable for advancing technology of nanowire-based systems suitable for sensor applications.

## Introduction

Semiconductor metal oxide nanomaterials have demonstrated a great potential for integration in a variety of devices, such as gas and humidity sensors, nanoelectronics, and low-power thermoelectrical generators [1–6]. Copper oxide (CuO) nanowires are excellent candidates for applications in such devices owing to the inexpensive, simple and scalable bottom-up synthesis, and robust physical properties [7–9]. A high specific surface area of nanowires and a p-type semiconductor structure are suggested for highly sensitive and fast responding nanowire-based gas sensors for the detection of CO, C_2_H_5_OH, H_2_S, and NO_2_ [10–14]. Unusually strong space-charge-limited currents observed in individual CuO nanowires [15] in combination with the mechanical strength [9,16] motivate their application as durable electrode interconnects for nanoelectronics. Specifically, they can be used to transduce electric signals in nanoelectromechanical system (NEMS) switches [5], which concerns the development of nanoelectronics capable to operate in harsh environments [17]. Additionally, the excellent thermal stability of CuO nanowires in combination with good electrical conductivity and thermoelectric power reaching 500 µV/K enables their application as p-type components for environmentally friendly thermoelectric devices [3–4].

Investigating the influence of relative humidity (RH) and understanding conductivity mechanisms in the assembled CuO nanowire networks is required for the technological development of such nanowire-based systems and to assess their operation possibilities in different environments. However, previous reports on humidity responses of nanostructured CuO systems are controversial. While in some reports, a decrease of conductivity in humid environments was observed, as expected for a p-type semiconductor material [6,13,18–19], other reports described a conductivity increase [20–24]. For example, arrays of free-standing nanowires showed an impedance increase upon exposure to humidity, which was explained by water chemisorption on the nanowire surface [6,13,18–19]. Nevertheless, for single nanowires assembled on electrodes by dielectrophoresis (DEP), the opposite response to humidity was observed [21]. Besides, unusual responses to humidity were shown for nanowires of other compositions aligned by DEP [25]. In some of these works, electrochemical impedance spectroscopy (EIS) was used as an effective tool to study the effects of chemical and physical absorption of water on the nanostructured surfaces of active CuO elements of the systems [23–24]. To the best of our knowledge, the effect of humidity on dielectrophoretically assembled CuO nanowire network systems with multiple interconnects, which may become very advantageous for the scalable assembly of CuO nanowire-based devices as NEMS [5], sensors [2], and thermoelectric modules [4], is yet to be reported.

In this work, CuO nanowires are synthesized by thermal oxidation [9] and aligned between metallic microelectrodes by DEP [26]. Electrical properties of the nanowire-based system at various RH values are assessed by EIS [27–28]. To attempt a systematic study on the suitability of the CuO nanowire networks for different applications, the measurements are performed in a RH range from 5% up to 97% and in a *T* range of 25–55 °C, which covers wider RH and *T* ranges than previous studies. Equivalent circuit models for the nanowire-based system are developed based on the EIS measurements. Fitting of the cell parameters shows contribution from resistance, capacitance, constant phase element, and Warburg element to the total impedance at various RH. Physical models describing the impact of chemisorption and physisorption processes are proposed to clarify the observed changes in the impedance spectra, discuss gas sensing mechanisms of nanostructured CuO, and indicate directions for further applications in humidity sensors and other systems with nanowire interconnects.

## Results and Discussion

**Figure 1:**
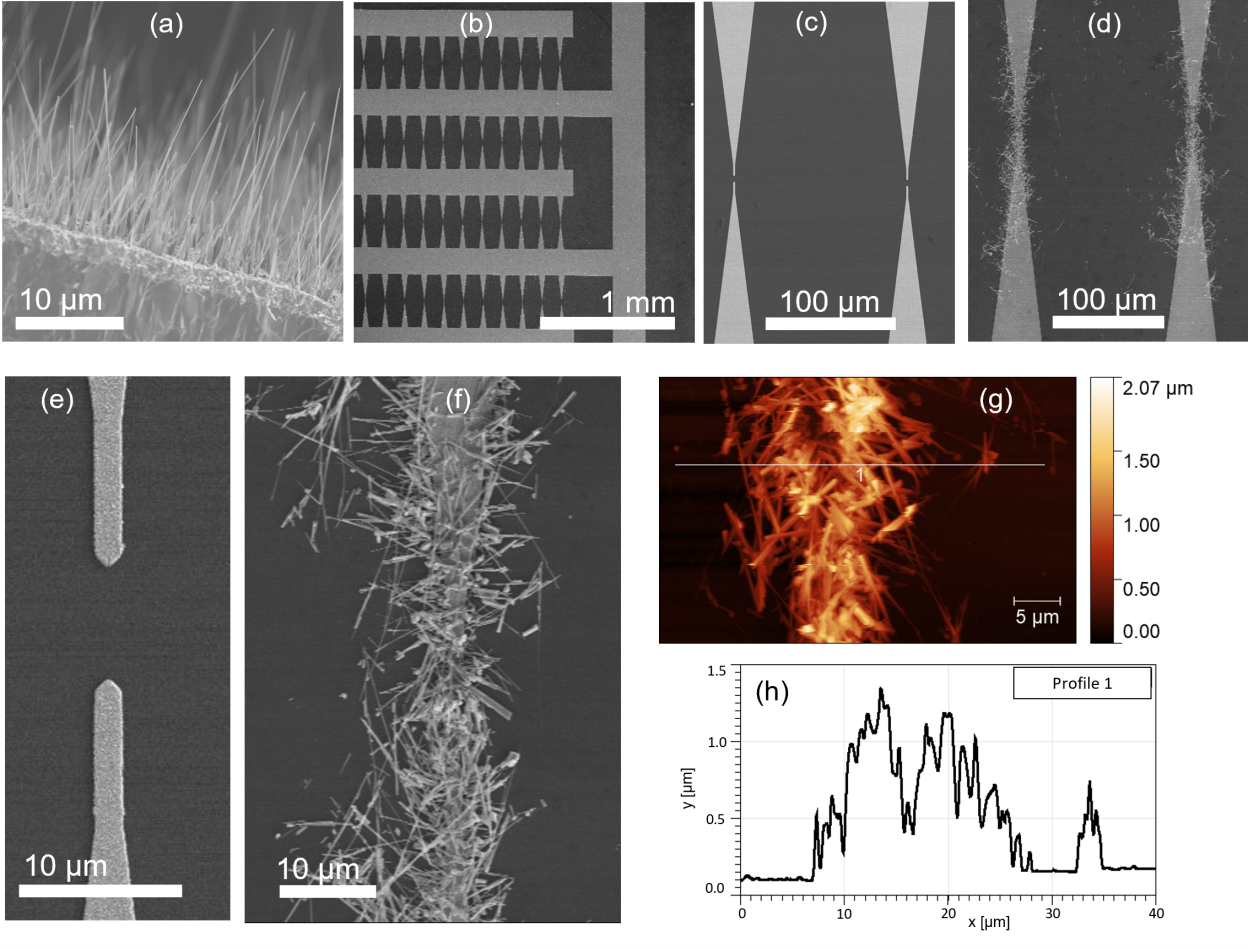
Scanning electron microscopy: (a) CuO nanowires grown on Cu substrate by thermal oxidation; (b) system of Cr/Au microelectrodes with teeth-like shape; (c–f) pairs of microelectrodes (c, e) without and (d, f) with dielectrophoretically aligned nanowires. Atomic force microscopy: (g) a bundle of CuO nanowires between microelectrodes and (h) a height profile scan across the bundle.

The synthesized CuO nanowires (Figure 1a) were assembled between arrays of lithographically defined Au microelectrodes on a Si/SiO_2_ chip (Figure 1b,c,e). The average diameters of the individual CuO nanowires, determined from the SEM images, were 50–100 nm, and their lengths varied between 2 and 20 µm (Figure 1a). To optimize the dielectrophoretic alignment of nanowires of such lengths and to maximize the number of the nanowire interconnects, the distance between the metallic microelectrodes was varied from 2 to 8 µm [26]. The arrays of microelectrode pairs with different gap distances were grouped in four rows on a chip (Figure 1b).

The dielectrophoretically aligned nanowires connected the gaps between pairs of microelectrodes, forming ordered and repeatable nanostructured interconnects (Figure 1d,f). The CuO nanowire bundles between the microelectrodes had typical widths of about 15–25 µm and heights of 1.0–1.3 µm (Figure 1g,h). The nanowires also covered the edges of the microelectrodes along approximately 100 µm distance from the gap (Figure 1d); however, the electrical signal was assumed to come from the CuO nanowire bundles interconnecting the electrodes.

**Figure 2:**
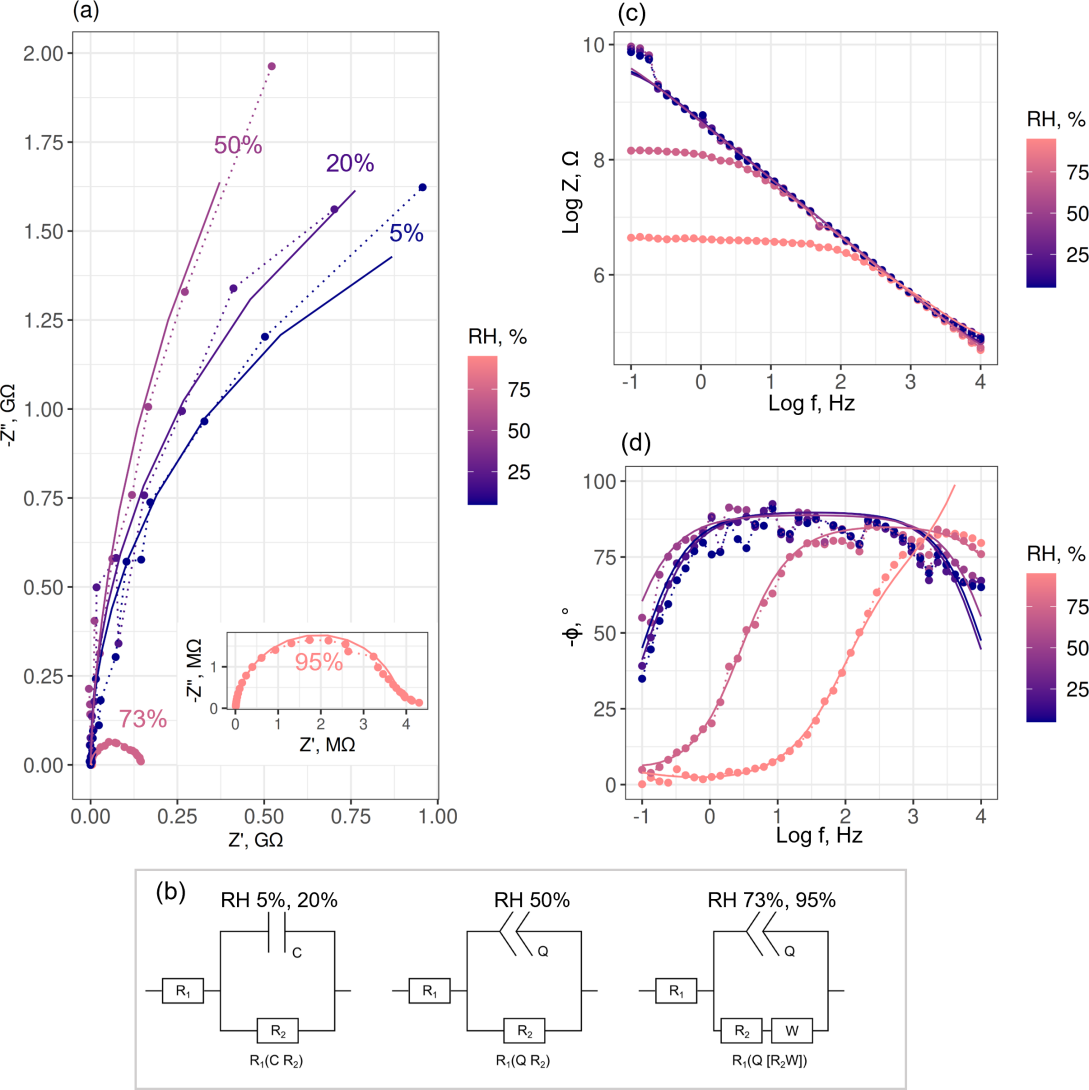
Impedance spectra measured for the system of CuO nanowire networks on microelectrodes at fixed *T* (30 °C) and various RH (5%, 20%, 50%, 73%, and 95%). Dotted lines connect measurement data points. Solid lines show the equivalent circuit fit results. (a) Nyquist plots: the frequency range shown in the graph is 0.24–10 000 Hz for RH 5–50%, 0.1–10 000 Hz for RH 73% and 95%; the inset shows a zoomed plot at RH 95%; (b) equivalent circuit models; (c, d) Bode plots of log *Z* vs log *f* and phase angle ϕ vs log *f*.

Examples of impedance spectra of the CuO networks interconnecting the metal electrodes are shown in Figure 2. Figure 2a shows Nyquist plots measured for variable humidity at a fixed temperature of 30 °C. As RH increases from 5% to 20% and 50%, the impedance increases; at higher RH (73%, 95%), the impedance decreases again. For the range of the measured frequencies *f* from 0.1 Hz to 10 kHz, the Nyquist plots have shapes of partial or full semicircles. At RH < 50%, the Nyquist plots can be described as parts of ideal circles with large radii, which correspond to the well-known circuit of a polarized electrode *R*_1_(*R*_2_*C*), where *R*_1_ is the resistance of a metallic electrode, *R*_2_ is the load resistance from the CuO nanowire interconnects, and *C* is the capacitance, which contains contributions from the water layer adsorbed on the nanowires, as well as from nanowire–nanowire and nanowire–electrode junctions (Figure 2b). The plots above RH 50% have the shapes of distorted circle parts, which denotes the presence of constant phase elements *Q* in the equivalent circuits (Figure 2b). The appearance of *Q* can also be deduced from the transition of log Z from a linear dependence to constant values in the Bode plots (Figure 2c). As the impedance decreases with the increase of RH, the semicircle shapes in the Nyquist plots become more distinguishable with smaller diameters (see, e.g., RH 73% and 95%, Figure 2a). A small tail at low frequencies (RH 95%, Figure 2a inset) corresponds to the appearance of a Warburg element *W* in the equivalent circuit (Figure 2b), which may be attributed to diffusion processes from liquids at high humidity.

The values of impedance magnitude 
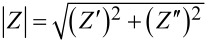
 and phase angle ϕ = tan^−1^(*Z*″/*Z*′) (where *Z*′ is the real part and *Z*″ is the imaginary part) are shown in Bode plots (Figure 2c,d). At RH levels below 50%, the log *Z* curves in the Bode plots (Figure 2c) show a linear dependence on log *f* in the complete frequency range and the impedance modules at low frequencies below 1 Hz, representing the ability of the sample to impede the flow of current between cathodic and anodic areas, equal to ca. 1 × 10^10^ Ω. When the RH is higher than 50%, the impedance modules at low *f* decrease by up to three orders of magnitude, and the log *Z* curves show a linear dependence on log *f* in the frequency range from 50 Hz to 10 kHz, followed by a transition to constant values in the lower frequency ranges (ca. 5 Hz for RH 73%, ca. 50 Hz for RH 95%). The phase angle in the Bode plots for RH ≤ 50% in the frequency range of 1–1000 Hz has a −ϕ value very close to 80° (Figure 2d). At higher RH levels, the phase angle at frequencies below 100 Hz (RH 73%) and 1000 Hz (RH 95%) decreases (Figure 2d). The decrease of the impedance module and the transition of log *Z* curves from a linear dependence to constant values, as well as the rapid decrease of the phase angle in the low-frequency region are related to the formation of a thicker and more disordered water layer at the CuO nanowire interconnects as discussed in the following paragraphs.

**Figure 3:**
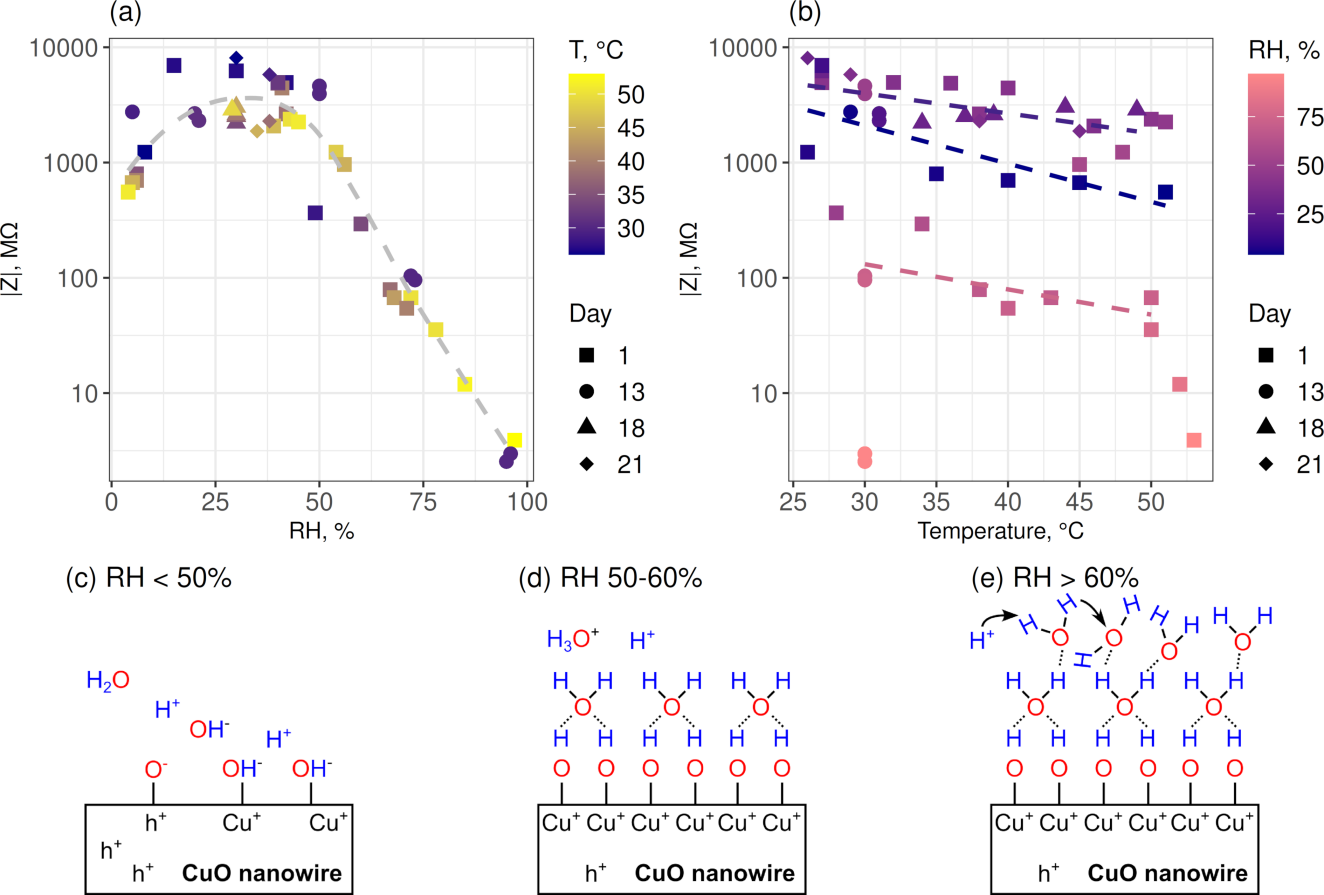
(a) Impedance magnitude |*Z*| vs relative humidity RH (dashed line is a guide to the eyes) at various *T*; (b) |*Z*| vs *T* at various RH. (c–e) Schematics of the processes induced by water chemisorption and physisorption on the CuO nanowire surfaces: (c) formation of a (Cu^+^–OH^−^) dipole layer chemisorbed on the surface of CuO nanowires with holes h^+^, and O^−^ ionosorbed from air at low RH, (d) H_2_O layer physisorbed atop of the OH^−^ layer, and (e) and proton hopping through the network of the physisorbed H_2_O molecules at high RH.

The total impedance magnitude |*Z*| of the CuO nanowire networks was found to vary as a function of RH and *T* in a wide range from a few megaohms to almost ten gigaohms. Figure 3a shows |*Z*| vs RH measured at various temperatures in one plot. The impedance is of the order of 500 MΩ at the lowest RH near 5% and increases up to approximately 2 GΩ at low RH levels near 10–20%. It remains high at RH levels up to 40–50% and then decreases by three orders of magnitude, when the RH increases from approximately 50–60% to 97%. The trend is supported by measurements after 13, 18, and 20 days (Figure 3a).

The impedance also decreased with increasing temperature, as it is expected for the semiconductor CuO [18]. The temperature dependence caused a variation of |*Z*| within one order of magnitude at similar RH (Figure 3b, dashed lines). Such variation of *Z* with the temperature is consistent with a previous report on a CuO sensor [18], where the measured resistance of a nanowire network system decreased by five times at a temperature rise from 20 to 80 °C. However, the *Z*–*T* dependence is less pronounced in our explored temperature region compared to *Z*–RH, where a change by three orders of magnitude (e.g., from 3 MΩ to 3 GΩ near 30 °C) across the range of explored RH can be observed (Figure 3b).

Such variations of the impedance with RH (Figure 3a) may be explained by parallel contributions to the net electrical signal from different conduction paths, for example, electronic and ionic transport inside the material and on the surface [29]. First, the conduction at very low RH is presumed to be primarily due to electronic transport inside the material. The CuO nanowires synthesized by thermal annealing of copper are a p-type semiconductor material [9,12,14]; its majority charge carriers are positive holes (h^+^) related to the presence of Cu vacancies or impurities. Under dry conditions, possible ionosorption of oxygen species from air on the nanowire surface adsorption sites (S), for example, in a reaction: 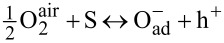 [30], may contribute to accumulation of holes near the surface (Figure 3c) [12,14,31]. Then, upon exposure to humidity, the conductivity reduces; impedances measured at RH 5–20% (ca. 0.5 GΩ) are lower than impedances of 2–8 GΩ measured in the RH region of 20–50% (Figure 3a). Possibly, at these RH levels, the concentration of free charge carriers in the CuO nanowires reduces, as hole trapping [32] occurs due to adsorption of gaseous H_2_O molecules on the CuO surface, dissociation in H^+^ and OH^−^, and formation of surface dipoles (Cu^+^–OH^−^). Water reactions with the adsorbed oxygen and Cu sites on the surface also neutralize holes: 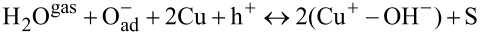 [30]. Hence, the impedance can increase with increasing RH (up to about 50% in our system) because of the affected electronic transport inside the material. Simultaneously, as the hydroxy groups begin to form clusters on the surface (Figure 3c), H^+^ hopping between neighbouring OH^−^ sites on the nanowire surface can be activated, which is an alternative conduction mechanism [29]. This ionic transport on the surface can compensate for the decrease of electronic transport conductivity inside the material. Since water chemisorption on the surface of a p-type nanowire can have a dual effect on the electrical conductivity, this may explain the scatter of impedances measured at the RH below 50% (Figure 3a). This hypothesis on the processes on the surfaces of the CuO nanowires is supported by the Bode plot data. The linear dependence of log *Z* on log *f* in the whole frequency range (Figure 2c) and the phase angle value of ca. 80° in the frequency range of 1–1000 Hz (Figure 2d) indicate that the electrochemical processes on the surface of the sample at RH < 50% result in a capacitive behaviour with good dielectric properties.

The decrease of the impedance down to a few megaohms starting at RH levels of 50–60% (Figure 3a) could mean an increasing dominance of ionic conductivity on the CuO surface. As the first (chemisorbed) OH^−^ layer on the nanowire surface becomes continuous (Figure 3d), a further increase in humidity causes physisorption of the second layer, that is, H_2_O molecules forming hydrogen bonds with the hydroxy groups [23,29,32]. This enables the formation of H^+^ and H_3_O^+^ ions, for example, when a proton is transferred from a hydroxy group to a water molecule [33]. A further increase of humidity up to ca. 90% causes the formation of additional physisorbed H_2_O layers through hydrogen bonding (Figure 3e). The conduction process occurs by the Grotthuss mechanism [29,33–34] of H^+^ hopping through the network of H_2_O molecules on the surface (H_3_O^+^ + H_2_O ↔ H_2_O + H_3_O^+^). Higher humidity causes increased concentration of H^+^ and hence more ionic conduction on the surface, which reduces the overall system impedance. For these RH levels, the Bode plots show a transition of the log *Z* vs log *f* curves from a linear dependence to constant values, as well as a rapid decrease of the phase angle from ca. 80° down to ca. 0° at higher frequencies in comparison with the Bode plots taken for RH 50% (Figure 2c,d), indicating an increase of thickness of the water layer.

Finally, the porous structure of the CuO nanowire bundles promotes capillary condensation of vapour, which can reach the substrate surface and enhance the conductivity through water droplets condensed on the surface at very high RH levels (e.g., near and above 90%). This is supported by the Bode plot data of the CuO nanowire interconnects taken for RH 95%, which showed a decreasing phase angle at frequencies below 1000 Hz (Figure 2d). This indicates that the condensed water reached the substrate.

Fitting of the impedance spectra across the examined range of RH was performed using equivalent electric circuits *R*_1_(*CR*_2_), *R*_1_(*QR*_2_) and *R*_1_(*Q*[*R*_2_*W*]) as mentioned above (Figure 2b). The capacitor impedance is *Z_C_*_,_*_Q_* = 1/[*Y*_0_(iω)*^n^*], where the angular frequency ω, the imaginary unit i, *Y*_0_ (*Y*_0_ = 1/|*Z*| at ω = 1 rad/s), and *n* (0 < *n* ≤ 1) do not depend on the frequency. For the capacitance *C*, *n* = 1, whilst for the constant phase element *Q*, *n* < 1. The impedance of the Warburg element is 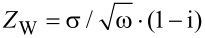, where σ is the Warburg constant.

**Figure 4:**
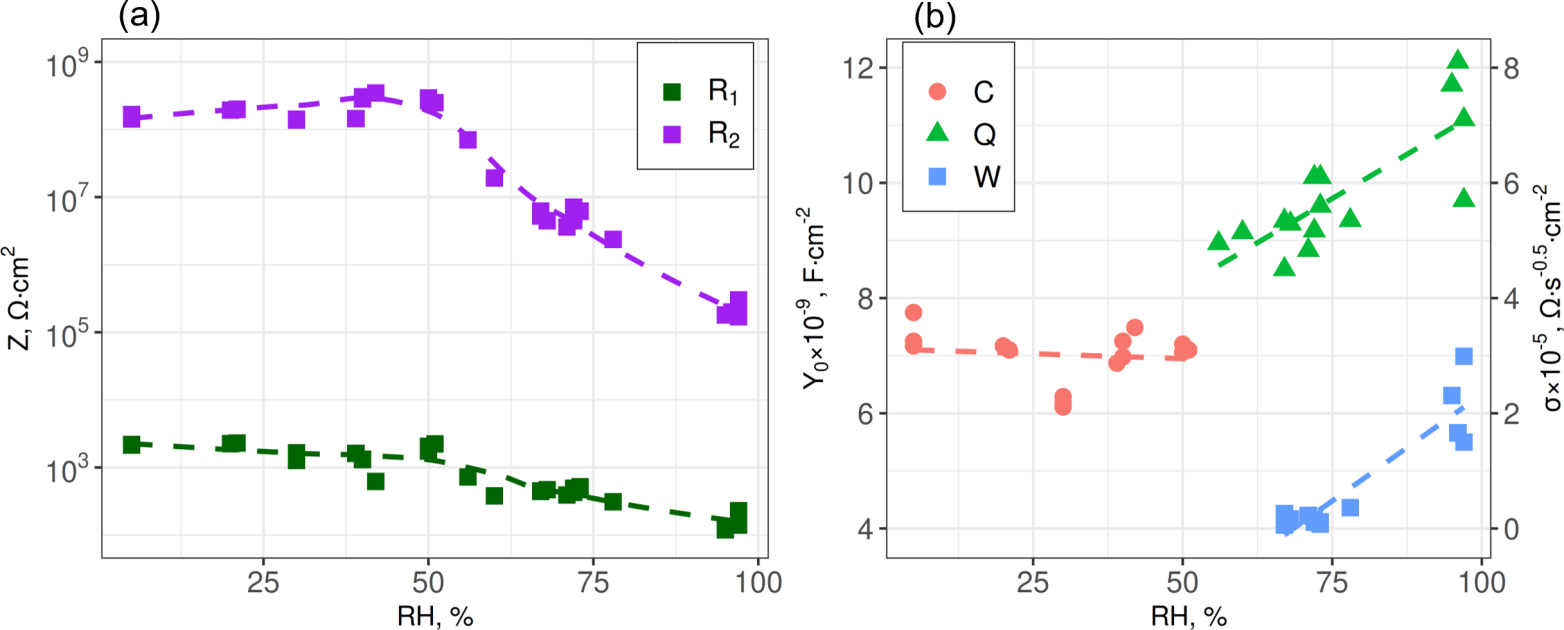
Normalized equivalent circuit parameters as functions of RH: (a) resistances *R*_1_ and *R*_2_ and (b) the Warburg constant σ and the parameter *Y*_0_ from the formula *Z_C_*_,_*_Q_* = 1/[*Y*_0_(iω)*^n^*]. For the capacitance *C*, *n* = 1, whilst for the constant phase element *Q*, *n* varied between 0.94 and 0.99 (e). Dashed lines are guides to the eyes.

At all RH, the resistance of a metallic electrode (*R*_1_) is much smaller than the load resistance *R*_2_ from the CuO nanowire interconnects (Figure 4a). An increase of *Y*_0_, which is a frequency-independent component of *C* or *Q*, with RH above 50–60% (Figure 4b) can mean that the polarization of water molecules on the surface of the nanowires becomes stronger when the thickness of the physisorbed water layer grows [27,32]. At RH above 60% (Figure 4b) the Warburg element appears and it grows with increasing the humidity, which suggests increased contribution of the diffusion processes to the total impedance with increased thickness of the water layer adsorbed at the nanowire interconnects (Figure 3e) and condensation of liquid water in pores.

The correlation of the equivalent circuit elements with RH can be analyzed to assess their suitability for potential application as sensor units. For example, *R*_2_ shows a more pronounced decrease with the increase of RH above 50% than *R*_1_ (Figure 4a). The humidity responses calculated as the ratio of maximal to minimal values 
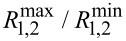
 are ca. 30 for *R*_1_ and ca. 2000 for *R*_2_. Also, the humidity response 
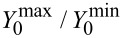
 of ca. 2 is even smaller than that for *R*_1_. All in all, the resistance *R*_2_ from nanowire interconnects is more suited for sensing because it changes with RH more prominently than the other elements of the equivalent circuits.

## Conclusion

In summary, EIS was applied to study changes in electric characteristics of a CuO nanowire-based system exposed to different humidity levels. Networks of CuO nanowires were assembled on microelectrodes by dielectrophoresis. The impedance of the device increased and remained high at RH up to 50–60% because of the chemisorption of water molecules on the surface of the p-type semiconductor nanowires. It decreased upon exposure to higher RH levels due to physisorption of water molecules atop of the chemisorbed layer and the increasing contribution of ionic conductivity in the water layers. At RH above 60%, the constant phase element and the Warburg element appeared in the equivalent circuit, suggesting an increasing part of water physisorption and condensation. All in all, the influence of these factors can explain the inconsistent humidity responses in CuO nanostructures and should be considered in further technological developments of CuO nanowire-based systems for sensing, nanoelectronic, and thermoelectric applications.

## Experimental

CuO nanowires were synthesized on Cu foil substrates (GoodFellow, 99.9% purity) by thermal oxidation [9]. The substrates were heated in air from room temperature to 500 °C for 30 min and maintained at the constant temperature for 210 min inside a GSL-1100X (MTI Corporation) quartz tube furnace. Then the heater was switched off, and the oxidized substrates cooled down naturally. The substrates were submerged in pure isopropanol (IPA) and ultrasonicated for 3 s to release the CuO nanowires.

These nanowires were assembled on arrays of Cr/Au (3/60 nm) microelectrodes lithographically pre-patterned on a commercially available Si/SiO_2_ wafer substrate (MTI Corporation) diced in 10 × 10 mm^2^ chips. To assemble the nanowires by dielectrophoresis, the chip was submerged in a suspension of the nanowires in IPA, and an AC voltage (5 V peak to peak, 5–50 kHz) was applied between the microelectrodes for 20 min [26]. Nanowires and microelectrodes were examined by scanning electron microscopy (SEM, Hitachi S4800) and atomic force microscopy (AFM, Asylum Research MFP-3D).

Electrochemical impedance measurements were performed under various atmospheric conditions in a custom-made system described elsewhere [28]. The main system parameters were RH from 4% to 97%, gas flow from 0.01 to 1.0 L/min, and temperature from 25 to 55 °C. Impedance spectra were measured using AUTOLAB PGSTAT 30. The impedance cell used for measuring the samples was grounded and protected from the surrounding electromagnetic radiation. Typical parameters for EIS measurements of CuO nanowires were an applied potential of 3–4 V, a current conditioning time of 5 s, a frequency in the range from 0.1 Hz to 10 kHz, and an amplitude (RMS) of 0.1 V. Before the measurements, the sample was held in constant RH for 20–25 min until the impedance spectra remained stable. The spectra were first measured in a nitrogen atmosphere and the RH was increased from 5% at the beginning of the measurements to 97% at the end by increasing the RH in steps of 5–10%; after that, the same was done in air atmosphere. No significant differences between the measurements in air and nitrogen were observed. Between the measurements, the sample was stored in air atmosphere at RH 20%.

The spectra were analysed using a complex plane Nyquist plot. The spectra were first fitted using the “Find circle” tool, and the equivalent electrical circuit was derived. Then the impedance element values were found using frequency response analysis (FRA) “Fit and Simulation 1.7”, which uses a non-linear least squares methods defined elsewhere [35]. For the impedance spectra, error values of the Kramers–Kronig complex test (Pseudo chi square) were in the interval from 1.55 × 10^−4^ to 1.0 × 10^−3^, “pseudo chi square real” was less than 1.0 × 10^−12^, and “pseudo chi imaginary” was less than 1.0 × 10^−10^, meaning that these spectra are valid for further analysis [36].

The EIS model of the whole tested sample consisted of 152 electrochemical cells, formed by nanowires interconnecting a pair of electrodes, connected in a parallel circuit. Using the nanowire bundle sizes and electrode gap distances, average electrochemical cell widths of 20 µm, cell heights of 1.1 µm and cell lengths of 7.2 µm were estimated. Numerical values for equivalent circuit parameters from fitting the experimental data were calculated per unit area considering the cell geometry and a parallel circuit scheme.
